# Funding and compliance to Test-Before-Treat recommendation in management of uncomplicated malaria among primary health care workers in Anambra State, Nigeria - a cross-sectional comparative study

**DOI:** 10.11604/pamj.2024.49.65.41337

**Published:** 2024-11-07

**Authors:** Uchenna Bridgid Chukwuka, Christian Chibuzo Ibeh, Prosper Obunikem Adogu, John Onuora Chukwuka

**Affiliations:** 1 Department of Community Medicine, Nnamdi Azikiwe University Teaching Hospital Nnewi, Anambra, Nigeria,; 2 Department of Community Medicine, Nnamdi Azikiwe University, Awka, Anambra, Nigeria,; 3 Department of Paediatrics, Nnamdi Azikiwe University, Awka, Anambra, Nigeria

**Keywords:** Malaria, Test-Before-Treat recommendation, compliance, primary healthcare centers, health workers

## Abstract

**Introduction:**

in 2010, Nigeria adopted the Test-Before-Treat recommendation in her “national guideline for diagnosis and treatment of malaria.” Across Nigeria, donor agencies including the Global Fund to Fight AIDS, Tuberculosis and Malaria, support states in malaria control, particularly at the Primary Healthcare level. This study aims to compare compliance to Test-Before-Treat recommendations in managing uncomplicated malaria among health workers in global fund-supported and government-supported Primary Healthcare Centers (PHCs) in Anambra State.

**Methods:**

a cross-sectional comparative study involving 32 PHCs across Anambra State. Using multi-stage sampling, the facilities were selected from four local government areas in two of the three senatorial zones. Data were collected using 1536 proforma for retrospective audit of case records, 82 health worker questionnaires, 32 facility assessment questionnaires, and 32 observational checklists for health facility inventory; and analyzed using IBM SPSS Statistics version 20. The Chi-square test of independence and Fisher’s exact test was used to determine the association between categorical variables. Statistical significance (p) was set at 0.05.

**Results:**

compliance with Test-Before-Treat recommendations in global fund-supported PHCs was 99% (760/768) versus 84% (645/768) in the government-supported (p=0.00). Comparatively, global fund-supported PHCs had higher availability of free malaria rapid diagnostic test kits (mRDT), job aids, copies of national guidelines in consulting rooms, staff with recent training on mRDT, and staff recently exposed to supervision.

**Conclusion:**

donor fund supports enhanced compliance to Test-Before-Treat recommendations by increasing the availability of free mRDT kits, job aids, National Guidelines, and frequency of staff exposure to supervision and training on mRDT.

## Introduction

To combat malaria, Nigeria, adopted the Test-Before-Treat recommendation in 2010 [1] in her “national guideline for diagnosis and treatment of malaria.” The Test-Before-Treat recommendation implies prompt parasitological confirmation of malaria by microscopy or rapid diagnostic test kit for all suspected cases before treatment is initiated [1]. The guideline further specified that treatment solely based on clinical suspicion should only be considered when a parasitological diagnosis is not accessible. Treatment based on parasitological confirmation improves the management of febrile illnesses and ensures that antimalarial drugs are used only when necessary [2].

Notably, malaria control in Nigeria has been largely donor-dependent. The global fund New Funding Model grant in Anambra was implemented from June 2015 to December 2017 [3,4]. The grant was implemented in 17 selected health facilities: 16 primary healthcare facilities and 1 Secondary Health facility, each in the 21 LGAs of Anambra State. Case management of malaria, both in health facilities selected for grant implementation (global fund-supported facilities) and in the rest of the health facilities in the state, government-owned (government-supported) and private, ought to be consistent with the recommendations of the national guideline for diagnosis and treatment of malaria.

However, studies done in Nigeria since the Test-Before-Treat recommendation was adopted, have demonstrated a persisting practice of presumptive treatment of fevers [5,6]. Also, literature on compliance of health workers to the Test-Before-Treat recommendation in Anambra State at the PHC level as well as literature on the impact of funding on compliance to Test-Before-Treat recommendation in the management of uncomplicated malaria (despite the huge donor dependency of malaria control in Nigeria), has been largely scarce. Hence, this study was designed to fill these gaps in knowledge; provide an evidence base for evaluation and future review of the malaria control program with or without donor funding; and thus, aid future planning of malaria control in the state and nation.

Study objectives: 1) to determine and compare the level of compliance to the Test-Before-Treat recommendation of the national guideline for diagnosis and treatment of malaria in managing uncomplicated malaria among health workers in global fund-supported and government-supported PHCs in Anambra State; 2) to identify and compare the factors affecting compliance to the Test-Before-Treat recommendation in managing uncomplicated malaria among health workers in global fund-supported and government-supported PHCs in Anambra State.

Null hypothesis: there is no difference in compliance with the Test-Before-Treat recommendation of the national guideline for diagnosis and treatment of malaria in managing uncomplicated malaria among health workers in global fund-supported and government-supported PHCs in Anambra State.

Alternate hypothesis: there is a difference in compliance with the Test-Before-Treat recommendation of the national guideline for diagnosis and treatment of malaria in managing uncomplicated malaria among health workers in global fund-supported and government-supported PHCs in Anambra State.

## Methods

Study design: this was a cross-sectional comparative study carried out from May to June 2018. It involved a questionnaire-based survey of health workers, a retrospective audit of records of patients treated for uncomplicated malaria from January 2016 to December 2017 (the global fund programme period); questionnaire-based health facility assessment; and health facility inventory in the selected Primary Healthcare Centers (PHCs) in each selected LGA.

Study location: Anambra State, Nigeria is an endemic malaria region with year-round transmission [7] and a malaria prevalence rate of 10.2% in children [8]. It has 4.18 million inhabitants [9], 3 senatorial zones, 21 Local Government Areas (LGAs), 330 wards and 177 communities [3]. Anambra has 1698 healthcare facilities: 1,090 private, 52 mission, and 556 government-owned (193 health posts, 329 PHCs, 8 Comprehensive Healthcare Centers, 24 secondary healthcare facilities (general hospitals), and 2 tertiary hospitals) [10].

Study population: there were 329 PHCs in Anambra State, 234 received global fund support for malaria control (global fund-supported/GFS PHCs) while 95 received no global fund support (government-supported/GS PHCs). Primary healthcare centers, dispensaries, and health posts are the first-level healthcare providers in the management of malaria [1]. The main form of malaria diagnosis at this level is the rapid diagnostic test kits. The core health workers in PHCs in Anambra State include nurses, Community Health Officers (CHOs), Community Health Extension Workers (CHEWs), and a few medical doctors. At the time of the study, there were about 896 core health workers at the LGA level in Anambra State. The medical doctors were not included in this study as they rotate through the GFS and the GS PHCs within the LGA of deployment.

Inclusion criteria: all the core health workers who worked in selected GFS or GS PHCs during the global fund programme period.

Exclusion criteria: the medical doctors in selected LGAs.

Sample size determination: the calculated minimum sample size for PHCs, health workers, and case records were 32, 80, and 1030 respectively using the following formula [11]:


$$n={\left\{u\surd [\pi_{1}(1-\pi_{1})+\pi_{0}(1-\pi_{0})]+v\surd [2\pi (1-\pi )]\right\}}^{2}÷{\left(\pi_{0}-\pi_{1}\right)}^{2}$$

Where, n= required minimum sample size of each group, π_1_= proportion of interest, π_0_= null hypothesis proportion, and π= (π_0_+π_1_) ÷ 2 (Annex 1).

Sample technique and sampling process: a multi-stage sampling was done. In the first stage, two senatorial zones out of the three senatorial zones in Anambra were selected by balloting. In the second stage, a simple random selection of two LGAs out of the seven in each selected Senatorial zone (using the list of LGAs in the Senatorial zone as a sampling frame) was done. In the third stage, in each selected LGA, all PHCs were stratified into GFS and GS study arms (using the lists of PHCs and GFS facilities in the LGA as sampling frames). Then, from each stratum, four PHCs were selected for the study by balloting resulting in eight PHCs in each selected LGA; 16 PHCs, in each selected senatorial zone; and 32 PHCs, in the state. In each selected PHC, all eligible health workers were recruited into the study.

Given the 1030 calculated sample size of case records, for this study involving 32 PHCs over 24 months, 1.34 case records per month per PHC were to be recruited. However, to eliminate fractions and yet attain the calculated minimum sample size, this was approximated to two case records per month per PHC giving a total sample of 1536 case records. This random sample was selected using a table of random numbers, with the general outpatient register as sampling frame.

Study instruments: a semi-structured questionnaire was self-administered to all eligible health workers in selected PHCs to assess self-reported practice in the diagnosis of uncomplicated malaria and also health worker-related factors affecting compliance to Test-Before-Treat recommendations. This instrument was adopted from three previous studies [6,12,13] and adapted to suit this study.

A health facility assessment questionnaire was also self-administered to the head of each selected PHC or a representative for identification of facility-level factors affecting compliance with the recommendation. This instrument was adopted from the Service Availability and Readiness Assessment (SARA) questionnaire [14] and adapted to suit the study. Using a Proforma in each selected health facility, data from case records of patients presenting with fever, and/or were either diagnosed with or treated for uncomplicated malaria from January 1, 2016, to December 31, 2017, were collected and audited to capture unbiased practices of health workers. This instrument was adopted from a previous study [15] and adapted to suit this study. A health facility inventory was undertaken using an Observational checklist to collect information from each facility´s inventory control card for December 2017 (provided by the head of each facility or a representative) on the availability and duration of stock-out of malaria diagnostic commodities. This instrument was adopted from the SARA questionnaire [14] and adapted to suit the study.

Study procedure: a community health extension worker was hired as a research assistant. A two-day training workshop was conducted to equip her with the necessary skills for conducting the survey, including understanding the study objectives, data capture tools, and the importance of good-quality data. Study instruments were pretested in a PHC center in an unselected LGA and observations made thereof informed revision of the final data capture tools where necessary. Data collection was done over 2 months during visits to selected health facilities. At the end of each day, the data collection tools were cross-checked for completeness, consistency, and legibility of the data collected. In case of missing or unreliable information, the source of the problem was identified and where necessary, the facility re-visited. The data collection tools were then stored in an orderly and safe manner.

Statistical analysis: data were entered into an Excel spreadsheet and analyzed using IBM SPSS Statistics version 20. Data analysis was done at the whole facility level (including GFS and GS) and then separately for each study arm. The variables were measured as proportions and presented in frequency distribution tables. Comparisons to determine associations were made using the Chi-square test of independence and the Fisher's Exact test. Statistical significance (P) was set at 0.05. Compliance with the Test-Before-Treat recommendation was measured as the proportion of case records with documentation of parasitological confirmation of malaria before the prescription of an anti-malarial drug. Any anti-malarial drug prescription without or before documentation of malaria investigation results in a case record was assumed to be presumptive treatment/treatment based on clinical evidence.

Factors related to health worker´s compliance with Test-Before-Treat recommendations were analyzed at health facility level (availability of malaria diagnostics) and health worker level (self-reported practice of health workers, exposure to training and supervision, health worker cadre, age of health worker, and availability of job aids) for each study arm and compared. The proportion of staff exposed to supervision within the last 6 months of 2017 was used as a measure of frequency of supervision; the proportion of at least a staff trained in mRDT within 2 years preceding the study as a measure of the frequency of mRDT training among health workers in the health facilities; availability of mRDT in December 2017 (the last month of the Global Fund Programme) as a measure of the availability of mRDT in the health facilities during the Global Fund Programme; and availability of mRDT on survey day (post Global Fund Programme) as a measure of availability of mRDT in health facilities when global fund support was non-existent.

Ethical considerations: approval for the study was obtained from the Nnamdi Azikiwe University Teaching Hospital Nnewi Ethics Committee, Anambra State Ministry of Health, the Health department of selected Local Government Areas, and heads of the selected PHCs. Individual informed consent (written) was also obtained from each participant in the study.

## Results

Data were collected from the retrospective audit of 1536 case records, 82 health worker questionnaires, 32 observational checklists for health facility inventory, and 32 health facility assessment questionnaires, in 32 PHCs in Anambra State. The demographic characteristics of the patients, sociodemographic characteristics of health workers, and the characteristics of the studied PHCs were as shown in Table 1 and Table 2.

**Table 1:** demographic characteristics of patients managed for uncomplicated malaria and sociodemographic characteristics of health workers in PHCs in Anambra State

Demographic characteristics of patients	GFS N=768; n (%)	GS N=768; n (%)	Test of significance	P-value
**Age categories**			χ^2^= 4.593; 1df	0.04
<5 years	287 (37.4%)	247 (32.2%)		
5 years or more	481 (62.6%)	521 (67.8%)		
**Gender**			χ^2^=1.643; 1df	0.22
Male	359 (46.7%)	334 (43.5%)		
Female	409 (53.3%)	434 (56.5%)		
**Sociodemographic characteristics of health workers**	**GFS N=41; n (%)**	**GS N=41; n (%)**	**Test of significance**	**P-value**
**Age categories**			χ^2^=0.760; 2df	0.68
40 years or less	7 (17.1%)	10 (24.4%)		
41-50 years	21 (51.2%)	18 (43.9%)		
51 years or more	13 (31.7%)	13 (31.7%)		
**Gender**			χ^2^=1.012; 1df	0.31
Male	0 (0.0%)	1 (2.4%)		
Female	41 (100.0%)	40 (97.6%)		
**Marital status**			χ^2^=1.810; 3df	0.61
Married	34 (82.9%)	34 (82.9%)		
Widowed	4 (9.8%)	2 (4.9%)		
Divorced	0 (0.0%)	1 (2.4%)		
Single	3 (7.3%)	4 (9.8%)		
**Cadre**			χ^2^=6.167; 2df	0.05
Trained nurse/midwife	17 (41.5%)	7 (17.1%)		
CHO	3 (7.3%)	6 (14.6%)		
CHEW	21 (51.2%)	28 (68.3%)		

GFS: global fund-supported, GS: government-supported, CHO: community health officer, CHEW: community health extension worker, χ^2^: Chi-square statistic

**Table 2:** characteristics of studied PHCs and facility-related factors affecting compliance of health workers to the Test-before-Treat recommendation in Anambra State

Facility characteristics	GFS (N=16); n (%)	GS (N=16); n (%)	Test of significance	P-value
**Availability of malaria diagnostic services**	16 (100%)	16 (100%)	N/A	N/A
**Malaria diagnostic method used in facility**			X^2^= 1.0; 1df	0.66
mRDT always	12 (75.0%)	12 (75.0%)		
Sometimes mRDT, sometimes microscopy/clinical judgment	4 (25.0%)	4 (25.0%)		
**Availability of trained malaria case management service providers**				
Any staff trained on mRDT in the past 2 years	10 (62.5%)	4 (25.0%)	X^2^ =4.751; 1df	0.03
**Availability of guidelines**			X^2^= 0.158; 2df	0.92
Observed available	3 (18.75%)	3 (18.75%)		
Reported available	6 (37.50%)	5 (31.25%)		
Not available	7 (43.75%)	8 (50.0%)		
**Availability of malaria diagnostic tools**				
**Any mRDT available in December 2017**			F.E =15.184; 1df	0.00
Reported available not seen	14/16 (87.5%)	3/16 (18.8%)		
Not available	2/16 (12.5%)	13/16 (81.2%)		
**Any mRDT available and valid on the survey day**			N/A	N/A
Observed	11/16 (68.75%)	11/16 (68.75%)		
Not available/reported available not seen	5/16 (31.25%)	5/16 (31.25%)		
**Microscopy not available**	16/16 (100.0%)	16/16 (100.0%)	N/A	N/A
**Stock-out of mRDT in December 2017**	2/16 (12.5%)	16/16 (100.0%)		
**Duration of mRDT stock-out**			F.E =0.132; 1df	0.72
14 days or less	0 (0.0%)	1 (6.2%)		
More than 14 days	2/2 (100.0%)	15/16 (93.8%)		
**mRDT stock-out within 4 weeks preceding the survey**	11/16 (68.8%)	10/16 (62.5%)	X^2^=0.139; 1df	0.71
**Duration of mRDT stock-out**			F.E =1.01; 1df	0.25
14 days or less	3/11 (27.3%)	1/10 (10.0%)		
More than 14 days	8/11 (72.7%)	9/10 (90.0%)		

GFS: global fund-supported, GS: government-supported, mRDT: malaria rapid diagnostic test kit, X^2^: Chi-square test statistic, FE: Fishers exact test statistic df: degree of freedom, guidelines: national guideline for diagnosis and treatment of malaria, N/A: not applicable, December 2017 represents the global fund programme period while the survey day represents a post Global Fund Programme period

The level of compliance to Test-Before-Treat recommendations among health workers in global fund-supported and government-supported PHC Centers in Anambra State: overall, 91.5% (1405/1536) of the studied case records had malaria investigation results recorded before anti-malarial drug prescription. Among the GFS PHC group, 99.0% (760/768) of case records had malaria investigation results recorded before anti-malarial drug prescription compared to 84.0% (645/768) of case records in the GS PHC group. There were 1% (8/768) of case records among the GFS PHC group versus 16% (123/768) of case records in the GS PHC group with no record of malaria investigation results but had antimalarial drug prescription (Table 3). The difference in the proportion of patients diagnosed based on parasitological versus clinical evidence between the 2 study groups was found to be statistically significant (Chi-square=110.367; P=0.00). Overall, 87.5% of the case records with evidence of malaria investigation had positive results (Table 3).

**Table 3:** health workers’ practice (as documented in case records) in diagnosis of patients presenting with fever, and/or either diagnosed with or treated for uncomplicated malaria from January 2016 - December 2017 in PHCs in Anambra State

Health worker’s practice	GFS N=768; n (%)	GS N=768; n (%)	Both groups N=1536; n (%)	P-value
**Mode of diagnosis**				0.00
Parasitological diagnosis	760 (99.0%)	645 (84.0%)	1405 (91.5%)	
Presumptive (clinical) diagnosis	8 (1%)	123 (16%)	131 (8.5%)	
**Parasitological diagnostic test used**				0.36
Malaria RDT	755 (99.3%)	643 (99.7%)	1398 (99.5%)	
Microscopy	5 (0.7%)	2 (0.3%)	7 (0.5%)	
**Malaria investigation result**				0.03
Positive	652 (85.8%)	578 (89.6%)	1230 (87.5%)	
Negative	108 (14.2%)	67 (10.4%)	175 (12.5%)	

GFS: global fund-supported, GS: government–supported, RDT: rapid diagnostic test, PHCs: public health care workers

Factors affecting compliance to Test-Before-Treat recommendations among health workers in global fund-supported and government-supported PHC Centers in Anambra State: several health worker-related factors could affect compliance with Test-Before-Treat recommendations including a cadre of health workers, length of practice, training of staff on the use of mRDT, staff training on microscopy, and staff training on the National Guidelines for Diagnosis and Treatment of Malaria (Table 1 and Table 4) were observed not to significantly differ between the 2 groups of health workers. However, percentage availability in the consulting room of (a measure of health worker´s access to) the national guideline for diagnosis and treatment of malaria and job aids (wall flow chart) was significantly higher among the GFS PHCs than among their GS counterparts (Table 4). Similarly, the proportion of staff exposed to supervision within the last 6 months of 2017 among the GFS PHCs was significantly higher than was observed among the GS group (Table 4). The self-reported practice of health workers in malaria diagnosis in each of the study groups is shown in Table 4. None of the variables assessing the health worker´s self-reported practice was found to significantly differ between the study groups.

**Table 4:** the self-reported practice of health workers and health worker-related factors affecting compliance to test-before-treat recommendation in PHCs in Anambra State

Health workers practice malaria diagnosis	GFS N=41; n (%)	GFS N=41; n (%)	Both groups N=82; n (%)	Test of Significance	P-value
**Length of practice**					
10 years or less	14 (34.1%)	8 (19.5%)	22 (26.8%)	χ^2^=2.353; 3df	0.50
11-20 years	17 (41.5%)	20 (48.8%)	37 (45.1%)		
21-30 years	8 (19.5%)	11 (26.8%)	19 (23.2%)		
31 years or more	2 (4.9%)	2 (4.9%)	4 (4.9%)		
**Staff training**					
Health workers trained on mRDT	32 (78.0%)	31(75.6%)	63 (76.8%)	χ^2^=0.069; 1df	0.79
Health workers trained in microscopy	0 (0.0%)	0 (0.0%)	0 (0.0%)	N/A	N/A
Health workers trained on the guideline	33 (80.5%)	37 (90.2%)	70 (85.4%)	χ^2^=1.562; 1df	0.21
**1^st^ training on the guideline**				χ^2^=1.855; 1df	0.28
8 years or less	30 (73.2%)	35 (85.4%)	65 (79.3%)		
9 years or more	11 (26.8%)	6 (14.6%)	17 (20.7%)		
**Number of trainings on the guideline**				χ^2^=0.892; 2df	0.64
One	12 (29.3%)	16 (39.0%)	28 (34.1%)		
Two	11 (26.8%)	10 (24.4%)	21 (25.6%)		
3 or more	18 (43.9%)	15 (36.6%)	33 (40.2%)		
**Preferred basis for diagnosis**				χ^2^=0.213; 1df	0.64
Clinical evidence	2 (4.9%)	3 (7.3%)	5 (6.1%)		
Parasitological evidence	39 (95.1%)	38 (92.7%)	77 (93.9%)		
**Suspected malaria cases tested**				χ^2^=0.703; 2df	0.70
All cases	32 (78.0%)	29 (70.7%)	61 (74.4%)		
Most cases	8 (19.5%)	10 (24.4%)	18 (22.0%)		
Some cases	1 (2.4%)	2 (4.9%)	3 (3.7%)		
**Health workers’ confidence in mRDT**				χ^2^=0.345; 1df	0.39
75%-100%	6 (14.6%)	8 (19.5%)	68 (82.5%)		
50%-74%	26 (63.4%)	26 (63.4%)	14 (17.1%)		
**Health workers’ perception of patients’ trust**				χ^2^=5.308; 2df	0.07
100% patient trust in mRDT	26 (63.4%)	17 (41.5%)	43 (52.4%)		
75% patient trust in mRDT	14 (34.1%)	19 (46.3%)	33 (40.2%)		
50% patient trust in mRDT	1 (2.4%)	5 (12.2%)	6 (7.3%)		
Demand antimalarial despite negative mRDT	37 (90.2%)	34 (82.9%)	71 (86.6%)	χ^2^=0.945; 1df	0.33
**Availability of guideline/job aid**					
Guideline in the consulting room	20 (48.8%)	11 (26.8%)	31 (37.8%)	χ^2^=4.201; 1df	0.04
The wall flow chart in the consulting room	37 (90.2%)	29 (70.7%)	66 (80.5%)	χ^2^=4.970; 1df	0.03
Any difficulty adhering to guideline	11 (26.8%)	7 (17.1%)	18 (22.0%)	χ^2^=1.139; 1df	0.29
**Any supervisory visit last 6 months of 2017**	13 (31.7%)	1 (2.4%)	14 (17.1%)	χ^2^=12.403; 1df	0.00

GFS: global fund-supported, GS: government-supported, guideline: national guideline for diagnosis and treatment of malaria, mRDT: malaria rapid diagnostic test, χ^2^: Chi-square test statistic

Facility-related factors that could affect compliance assessed include availability in the health facility of at least a staff trained in mRDT within 2 years preceding the study (a measure of the frequency of training) and of mRDT in December 2017 (the last month of the global fund Programme) (Table 2). Both factors were found to be significantly higher among the GFS PHCs compared to the GS group. Availability of mRDT on survey day (Post global fund programme) was also assessed (Table 2) and found not to significantly differ between the two groups.

## Discussion

The overall compliance of health workers to the Test-Before-Treat recommendation of the National Guidelines for Diagnosis and Treatment of Malaria was observed to be 91.5%. For the study groups, the compliance of health workers in GFS PHCs was observed to be higher (99%) compared to those in GS PHCs (84%). This was similar to their self-reported practice in which 93.9% overall, 95.1% in the GFS group, and 92.7% in the GS group preferred parasitological evidence as the basis for malaria diagnosis. The observed compliance, overall and among each group was higher than was observed in some previous studies [6,16] Also, a study in Senegal reported 86% compliance which is lower than was observed among health workers in this study overall and for the GFS PHC group but is higher than was observed among health workers in the GS PHC group [17]. The high level of overall compliance of health workers to the Test-Before-Treat recommendation observed in this study compared to earlier studies may be due to improved knowledge, awareness, acceptance, and behaviour change among health workers, towards the Test-Before-Treat recommendation seven years post-adoption.

Concerning factors affecting the compliance of health workers to Test-Before-Treat recommendations, the GFS group was observed to have a higher proportion of health workers exposed to frequent supervision; and a higher proportion of facilities with available mRDT kits, with at least a staff recently trained on mRDT and in which malaria case management job aids and National Guidelines for Diagnosis and Treatment of Malaria were both available and accessible to the health worker during the global fund programme, compared to the GS group. Some previous studies reported the availability of free mRDT [12,17-21], health worker training on mRDT [18,22], poor/infrequent supervision [22] and guideline availability/emphasis [20] as factors affecting compliance as was observed in this study.

Some other studies however reported knowledge of mRDT [18,19,21] trust in mRDT [12,16,18,21] preference for other methods [19], staff shortage [12], limited capacity to diagnose other causes of fever [12], patient expectations [16], work experience (length of practice) [16], and health worker cadre [16] as factors affecting compliance. Most of these factors were accessed in this study and found not to influence compliance thus further suggesting, that there was indeed improved knowledge, awareness, acceptance, and even practice of the Test-Before-Treat recommendation among health workers. However, the level of compliance appears to be largely dependent on the availability of material resources (mRDT, job aids, and guidelines) and the frequency of human resource (health worker) training and supervision. It is particularly noteworthy, that the aforementioned Senegal study [17] preceded the introduction of the Test-Before-Treat recommendation by the World Health Organization in 2010, yet a nationwide increase in the availability of mRDT markedly increased parasitological confirmation of malaria from 3.9% to 86% over 2 years. Thus, indicating that increasing the availability of mRDT alone enhances parasitological confirmation of malaria and hence compliance with Test-Before-Treat recommendations.

**Limitations of the study:** it is noteworthy that some of the findings in this study were based on a retrospective audit of records (case records of patients and inventory control cards) and hence were dependent on the accuracy of record keeping.

## Conclusion

In this study, a high level of compliance to the Test-Before-Treat Recommendation by health workers in both study arms was observed with a higher compliance rate among the global fund-supported facilities. The higher level of compliance among the GFS PHCs may be attributed to the higher availability of free mRDT kits; staff with recent training on mRDT; malaria case management job aids (wall flow chart) and national guideline for diagnosis and treatment of malaria in the consulting rooms (accessible to the health workers). This difference in compliance with the Test-Before-Treat recommendation in the management of uncomplicated malaria among health workers in GFS and GS PHCs in Anambra State hence favours the rejection of the null hypothesis.

The areas of strength (observed to have enhanced compliance to the Test-Before-Treat recommendation in the donor-supported PHCs) are to be consolidated and areas of weakness, be constructively reviewed in the future planning of the Malaria control programme in Anambra State. This study has thus provided evidence for the evaluation and replanning of the Malaria control programme in Anambra State and Nigeria. Hence, to enhance compliance of health workers to the Test-before-Test recommendation of the National guideline in the case management of uncomplicated malaria there is need to increase and sustain supply of free malaria diagnostic commodities (mRDT kits), ensure a universal supply of malaria case management job aids (wall flow charts) and National Guidelines for Diagnosis and Treatment of Malaria to all PHCs in Anambra State while ensuring 100% access to these materials by the health workers, for more regular supervision of the health workers in PHCs and training and retraining of health workers in PHCs on the Test-before- treat recommendation of the national guideline for diagnosis and treatment of malaria.

### What is known about this topic

- Nigeria adopted the Test-Before-Treat recommendation in 2010 in her “national guideline for diagnosis and treatment of malaria” to combat Malaria;
- Studies done in Nigeria since the adoption of the Test-Before-Treat recommendation have demonstrated a persisting practice of presumptive treatment of fevers.

### What this study adds

- In this study, a high level of compliance to the Test-Before-Treat Recommendation by health workers in primary healthcare facilities in Anambra State, with a higher compliance rate among the global fund-supported facilities, was observed;
- Donor fund support enhanced compliance to Test-Before-Treat recommendations by increasing the availability of free mRDT kits, job aids, National Guidelines and frequency of exposure of health workers to supervision and training on mRDT.

## Annex

**Annex 1:** sample size determination (PDF - 120 KB)
